# Personalized ECT: Much ado about nothing?

**DOI:** 10.1192/j.eurpsy.2021.84

**Published:** 2021-08-13

**Authors:** P. Sienaert

**Affiliations:** Academic Center For Ect And Neuromodulation (accent), University Psychiatric Centre KU Leuven, Kortenberg, Belgium

**Keywords:** Electroconvulsive therapy, electrode position, personalized medicine

## Abstract

The discussion about whether or not to focus our attention on the ‘average’ ECT-treatment technique that suits the majority of our patients or tailor the treatment to the needs of individual patients is ongoing. The question is, however, whether the available evidence permits us to to offer treatment ‘à la tête du client’. The start of a treatment course can be personalized by choosing electrodeplacement (EP) (e.g. bilateral in case of a severe or life-threatening condition, when fast improvement prevails over cognitive impact), parameter selection (e.g. a shorter pulse-width in order to avoid cognitive side-effects), and a dosing strategy. A fixed-dose will lead to overdosing in some patients (causing side-effects) and under-dosing in others (delaying/decreasing response) (1) Adjusting an ongoing treatment-technique can be based on response, side-effects or on the quality of the elicited seizure (EEG). In case of inadequate response, the clinician can decide to switch EP or to increase dose. There is no consensus as to the number of sessions after which technique should be changed. In case of intolerable side-effects, parameter selection and/or EP can be adjusted. The evidence that is available to guide these steps is limited. There is some evidence for a relation between several EEG-characteristics and outcome. Thus, in the event of an inadequate seizure, changing the anesthetic regimen, optimizing ventilation, lengthening the anesthetic-ECT time-interval or increasing the stimulus dose, can be of help. 1. Sackeim et al. Treatment of the modal patient: does one size fit nearly all? J ECT 2001;17:219-222.

**Disclosure:**

No significant relationships.

